# Towards a methodology for cluster searching to provide conceptual and contextual “richness” for systematic reviews of complex interventions: case study (CLUSTER)

**DOI:** 10.1186/1471-2288-13-118

**Published:** 2013-09-28

**Authors:** Andrew Booth, Janet Harris, Elizabeth Croot, Jane Springett, Fiona Campbell, Emma Wilkins

**Affiliations:** 1 School of Health and Related Research (ScHARR), University of Sheffield, Regent Court, 30 Regent Street, Sheffield S1 4DA, UK; 2 Centre for Health Promotion Studies, School of Public Health, University of Alberta, 3-289 Edmonton Clinic Health Academy 11405–87th Ave Edmonton, AB T6G 1C9, Edmonton, Canada

**Keywords:** Bibliographic databases, Database searching, Literature searching, Search strategies, Systematic reviews

## Abstract

**Background:**

Systematic review methodologies can be harnessed to help researchers to understand and explain how complex interventions may work. Typically, when reviewing complex interventions, a review team will seek to understand the theories that underpin an intervention and the specific context for that intervention. A single published report from a research project does not typically contain this required level of detail. A review team may find it more useful to examine a “study cluster”; a group of related papers that explore and explain various features of a single project and thus supply necessary detail relating to theory and/or context.

We sought to conduct a preliminary investigation, from a single case study review, of techniques required to identify a cluster of related research reports, to document the yield from such methods, and to outline a systematic methodology for cluster searching.

**Methods:**

In a systematic review of community engagement we identified a relevant project – the Gay Men’s Task Force. From a single “key pearl citation” we conducted a series of related searches to find contextually or theoretically proximate documents. We followed up Citations, traced Lead authors, identified Unpublished materials, searched Google Scholar, tracked Theories, undertook ancestry searching for Early examples and followed up Related projects (embodied in the CLUSTER mnemonic).

**Results:**

Our structured, formalised procedure for cluster searching identified useful reports that are not typically identified from topic-based searches on bibliographic databases. Items previously rejected by an initial sift were subsequently found to inform our understanding of underpinning theory (for example Diffusion of Innovations Theory), context or both. Relevant material included book chapters, a Web-based process evaluation, and peer reviewed reports of projects sharing a common ancestry. We used these reports to understand the context for the intervention and to explore explanations for its relative lack of success. Additional data helped us to challenge simplistic assumptions on the homogeneity of the target population.

**Conclusions:**

A single case study suggests the potential utility of cluster searching, particularly for reviews that depend on an understanding of context, e.g. realist synthesis. The methodology is transparent, explicit and reproducible. There is no reason to believe that cluster searching is not generalizable to other review topics. Further research should examine the contribution of the methodology beyond improved yield, to the final synthesis and interpretation, possibly by utilizing qualitative sensitivity analysis.

## Background

As systematic review methodologies seek to incorporate an ever wider variety of types of evidence, and to integrate both quantitative and qualitative data, review teams need to develop ever more innovative and imaginative techniques of synthesis [1,2]. Innovative methods of synthesis, in turn, require that the team moves away from reliance on topic-based search techniques that are specified *a priori* towards more creative, intuitive and iterative procedures for evidence identification [3]. Such exacting demands are exemplified by systematic reviews of complex interventions.

Emerging systematic review methods for complex interventions often seek to identify underpinning theories to explore, and attempt to explain, what exactly is happening as a result of the intervention [4]. In addition to this explanatory function theories may be used for a more instrumental purpose – to construct a framework by which reviewers extract and subsequently analyse data from included studies [5]. A theoretical framework may therefore act as either a “window” for illumination and/or as a “scaffold” for construction of the review (Table 1).

**Table 1:** Systematic review methodologies requiring identification of theory and/or context

**Methodologies requiring identification of theory**	**Methodologies requiring identification of context**
Best fit framework synthesis	
Framework synthesis	
Realist synthesis	Realist synthesis
Systematic review of complex interventions	Systematic review of complex interventions

Systematic review methods for complex interventions also typically require a review team to gain an in-depth understanding of context and of implementation issues. The team needs to identify “thick” data to enable them to explain not simply “what works” but 'what works for whom, in what contexts, and why’ [6] (Table 1). Teams at the EPPI-Centre (Institute of Education, University of London) conduct separate reviews of outcome studies (e.g. randomized controlled trials) and process evaluations and then interpret the findings using a technique known as narrative synthesis [7]. The Cochrane Collaboration similarly seeks to enhance its systematic reviews of effects by undertaking syntheses of qualitative research [8].

Such a move poses at least two major challenges to identification of relevant evidence: first, search methodologies must be sufficiently robust and rigorous to preserve the credibility of the review, and, second, iterative and intuitive search procedures may render it problematic “to use completely reproducible and transparent search and selection strategies” [9]. Few existing search strategies manage to be rigorous, robust, reproducible and transparent while remaining iterative and intuitive. Systematic review search methodologies, for example the use of study filters, perform well against the former requirements by being necessarily “controlled” against deviation from the protocol. Traditional search techniques may offer flexibility to accommodate iterative approaches but may do so at the expense of being reproducible and transparent. These extremes may be caricatured on a continuum that extends from viewing information retrieval as a science through to considering searching to be an art [10]. Might it be possible to develop methods of evidence identification that may be both iterative and systematic?

Many commentators would add *comprehensive sampling* to the defining characteristics of searches in support of systematic reviews. However recent developments in systematic review methodology make this a contested area [11,12]. While systematic omission of relevant evidence, and its associated biases, is anathema for any systematic review, reviewers increasingly acknowledge that it is the appropriateness of the sample, not its comprehensiveness, that is the critical factor [13]. Different emerging review methodologies harness such sampling methods as theoretical sampling (e.g. realist synthesis), snowball sampling (e.g. meta-narrative approaches) and maximum variation sampling (e.g. framework synthesis) [14]. Interpretative reviews seek to acquire a holistic understanding of a phenomenon but may well reach a point of theoretical saturation through purposive sampling where no further insights would be added by a comprehensive sampling approach [15]. Random sampling has been explored in the specific context of scoping reviews [13]. This variety of possible sampling methods places three particular imperatives on a review team; they must select their sampling method appropriately, they must seek to communicate to their reader why their chosen sampling method is appropriate and they must select a search method that carries the potential to achieve their chosen sampling approach. Where these three imperatives are satisfied a review may indeed possess the systematic review characteristics of being systematic, transparent and reproducible.

In a seminal information retrieval paper Bates described a set of techniques termed 'berrypicking’ [16], where follow up of initial searching against a broad topic leads to further ideas and directions. “Berrypicking” is a leading example of a traditional search technique that predates the development of systematic review methods. Subsequently it has been harnessed only selectively in the context of systematic reviews. Rather than sticking to an *a priori* search protocol Bates described how a searcher's concept of a query is influenced by every new item of information that they encounter. A useful reference may suggest a particularly fruitful line of inquiry, either suggesting a need to graze further around a particular source or, if experiencing diminishing returns, to move on to pastures new. Employing the metaphor of berry gathering, search queries are typically neither static nor linear, but rather iterative, evolving as new information becomes available.

Berrypicking has recently been recommended in the context of knowledge building and theory generating qualitative systematic reviews [17]. However it poses particular challenges due to perceived deficiencies with regard to limitations in systematicity, transparency and reproducibility [18]. In addition berrypicking causes particular anxiety for any who wrongly associate such approaches with being haphazard, ill-disciplined and amateurish.

Although well-established, the berrypicking approach now commands particular attention as a potential first-line procedure for systematic reviews, as opposed to previously being conceived as a “safety net”. This coincides with ongoing refinement of what exactly is meant by “systematic” [19,20] within the context of the label of “systematic review”. A reevaluation of the value of berrypicking is particularly timely given increasing recognition of the importance of context, a factor poorly catered for by topic-based bibliographic searching.

This paper aims to explore whether it is possible to develop an explicit methodology for the identification of conceptually rich or contextually thick “clusters” of data (abbreviated as “cluster searching”), to help explore the theoretical underpinnings and/or the context for a complex intervention. It seeks to systematize, and thus extend, accepted use of the “berrypicking” methodology within systematic reviews to identification of a cluster of related reports. These possibilities are explored within an individual case study, presented as a narrative to illustrate the value of a cluster-based approach, within a National Institute for Health Research-funded project entitled, Community-based peer support: Developing a model for promoting health literacy (COPES).

### Berrypicking

Six techniques were highlighted by Bates as a means to harvest additional information: footnote chasing (backwards chaining from articles of reference, tracking back footnotes), citation searching (forward chaining, using a citation index to jump forward), journal run (using authoritative journals on a subject and going through the entire run), area scanning (using the physical location or layout of a resource on the assumption that relevant materials will be co-located), abstracting and indexing searches (using organized bibliographies and indexes, usually arranged by subject area) and author searching [16]. Berrypicking has been used extensively for those types of review where exhaustive searching is not formally required, such as qualitative metasynthesis [3,21-24]. Indeed Walsh and Downe (2005) challenge the appropriateness of the *a priori* protocol-based search strategy used for quantitative systematic reviews [25], where the search strategy is fully formed before formal searching begins. They state that such an approach is only valid if it leads to a linear process of “decision-description-search-location”. They contrast a protocol-based approach with Bates’ 'berrypicking model’ which allows for search directions to be divergent rather than linear. Under such circumstances berrypicking utilises the purposive sampling approach that characterises primary qualitative research.

One might counter criticisms of the protocol *per se* by arguing that prespecification of search methods in a protocol seeks to contribute procedural objectivity [26]. While this criterion may not always be appropriate, depending upon the degree to which an individual review avows to be interpretative, protocols may also have additional utility beyond this – as a planning and communication tool. As a statement of intent they provide a valuable focus for feedback and input into the review from a wider audience. However it is important not to confuse these inherent advantages, including the requirement to give advance consideration to potential issues, with the controlling out of all facility to be iterative, intuitive and to have the potential to “follow up” productive leads. A protocol, while prespecifying the types of sampling and searching that will take place and satisfying the reader or commissioner of the review that these are appropriate, does not necessarily have to prescribe the exact nature of all procedures.

### Identifying theories

The UK Medical Research Council Framework for the Development and Evaluation of Complex Interventions (2008) specifies “establishing a theoretical basis” as Stage 1 in the development of any complex intervention [27]. It may be considered similarly important when undertaking a systematic review to evaluate such an intervention. The MRC guidance suggests involvement of experts, other stakeholders and the use of qualitative research in identifying relevant theory. Little attention is paid to the identification of theory through systematic search procedures, not least because such procedures are not known to exist. While it is undesirable to restrict the subsequent analysis by accessing theories inappropriately or indiscriminately it is clearly equally problematic to attach too great a significance only to theories already known to experts or other stakeholders within a particular project. Indeed systematic identification of theories from the literature could arguably access a wider range of candidate theories from experts, other stakeholders and from qualitative research from a group of related projects than otherwise available from the corresponding sources within a single project.

Several factors related to authors’ use of theory combine to require that the search for relevant theory is as persistent and wide-ranging as time and review resources allow. Some authors only use theory superficially to provide credentials for their choice of intervention, others use a particular theory imperfectly such that it bears little resemblance to its origins, still others provide little detail of the theory (or omit it all together) through word limitations or the publishing conventions of their particular discipline. None of these limitations invalidates a systematic search for theory per se. However they do emphasise that results must be handled with caution.

#### On conceptual richness

Ideally a report of a complex intervention should not only adequately describe the intervention and its context (“contextual thickness”) but should also possess “conceptual richness”. Our working definition of conceptual richness encompasses “a degree of theoretical and conceptual development that explains how an intervention is expected to work”. Conceptual richness is typically evoked in a systematic review context when undertaking meta-ethnography [28], which seeks to generate theory, or realist synthesis, which seeks to explore and test theory [29]. However conceptual richness is equally important in the context of complex interventions, as the working definition signifies. Ideally a randomized controlled trial would describe the theoretical underpinnings of the intervention being evaluated. More frequently, however, the theoretical content is detached from the trial, being located in an associated publication or in an early study of which the identified study is a derivative. Hence a review team will need to consider the use of systematic techniques of cluster searching.

### Exploring context

A review team would ideally acquire an understanding of context by examining studies that have been conducted alongside an effectiveness study [4]; either as part of an integrated mixed methods study or as a “sibling study” [30] (Table 2). Sibling studies may include qualitative research studies, economic evaluations or process evaluations associated with specific randomised controlled trials. Such studies are particularly valuable because they are commissioned specifically to explore the context surrounding an effectiveness study, with the explicit aim of documenting the process and explaining contextual factors that influence implementation and/or outcomes.

**Table 2:** Terminology associated with cluster searching

**Concept**	**Description**
Cluster searching	A systematic attempt, using a variety of search techniques, to identify papers or other research outputs that relate to a single study. This relation may be direct (i.e. “sibling” papers produced from the same study) or indirect (“kinship” studies that inform theoretical or contextual elements of the study of interest).
Key pearl citation	A key work in a topic area, specifically in this context a report of a research study that acts as a retrieval point for related outputs that may help to explicate theory or to understand context.
Kinship study	A study subsequently identified as being related to an original study of interest. Kinship studies may share a common theoretical origin, links to a common antecedent study or a contemporaneous or spatial context.
Sibling paper	A paper subsequently identified as being an output from the same study as an original paper of interest.
Study cluster	A group of inter-related papers or other research outputs that relate to the same single research study.

#### On contextual thickness

Limitations on reporting placed by individual journals and their respective guidelines further constrain data on the context for an intervention. Randomised controlled trials are expected to adhere to the extensive CONSORT publication standards leaving little room for a detailed description of context [31]. Typically trials provide only a brief description of “Setting”. In contrast, those studies that contribute most to understanding of an intervention or service will possess greater “thickness” of detail [32,33]. A thick description has four different attributes. It “(1) describes the context of an act; (2) it states the intentions and meanings that organize the action; (3) it traces the evolution and development of the act; (4) it presents the action as a text that can then be interpreted. A thin description simply reports facts, independent of intentions or the circumstances that surround an action (p. 33)” [34].

Contextual thickness can be seen to require:

1. Sufficient detail to enable the reader to establish what exactly is going on, both associated with the intervention and associated with the wider context.

2. Sufficient detail to enable the reader to infer whether the findings can be transferred to other people, places, situations, or environments [35].

Such thickness is unlikely to be present within a single report of a study published in the peer reviewed journal literature [36]. Instead a review team will need to move away from the individual paper towards the “study cluster” – that is all reports, published or unpublished, that may directly inform the specific context, or indirectly, illuminate the theoretical ancestry, of the study in question. The study cluster may include quantitative and qualitative research, grey literature reports to supplement formal published literature, and may include informal types of data (such as information from project web pages) as well as theory papers associated with the intervention. It may further include data on cost effectiveness, from published studies or from accompanying technical reports. Such a cluster will expand longitudinally throughout the life of the study. Relevant study reports may include preparatory information from the study protocol or from a preexisting needs assessment. They may also extend beyond the life of the project to reports of long term follow up or critiques and commentaries of the project and its associated papers. Taken individually each data source would be judged differently regarding its scientific *rigour* and *external validity*. It is therefore likely that quality assessment would have to be performed using the complete study as the unit of analysis, rather than at the level of an individual paper or study report. This requires further investigation. Taken as a body of evidence, however, and privileging contextual *relevance*, the collective accounts offer a value-added contribution to the phenomenon under study.

#### When “direct evidence” is lacking

Where direct evidence from sibling studies does not exist there may still be value in retrieving studies from a common context (e.g. they might be contemporaneous and within the same country, despite being in different localities). Here a review team assumes that shared characteristics of the associated studies – for example locality, population demographics, conditions, and experiences - can provide indirect insights. A qualitative study examining the context of a specific intervention in, say, Bristol (in South West England) has the potential to illuminate how that same intervention was seen to work in a randomized controlled trial performed in Newcastle (in North East England) [37]. Such an affinity equates more to “kinship” (Table 2), particularly when contrasted with the direct comparisons offered by sibling studies (Additional file 1 Schema of Cluster Documents). At a review level the EPPI-Centre method requires the “judgment of reviewers when evaluating the extent to which an intervention meets a recommendation from the qualitative synthesis” [38], particularly so if the latter evidence base is derived from unrelated process evaluations.

Finally where there is a focus on mid-range theories, rather than on specific interventions, a review team may seek to derive value from a loose collection, or bundle, of qualitative studies conducted across a variety of temporal and spatial settings with different populations or disease groups. Under such circumstances the diversity of the sample of qualitative studies [39], may illuminate the mechanisms of action of an intervention in an equally divergent group of randomized controlled trials conducted within an equally varied range of settings. Such an approach argues, for example, that the range of mechanisms by which performance league tables “work” for hospitals might include some, but by no means all, of those mechanisms that have utility in explaining why and how similar league tables “work” for schools [40].

### Identifying study clusters

There is little published guidance on how to identify and retrieve a “study cluster”. In particular there is little empirical work associated with the characteristics of sibling studies. The emphasis of the Community-based peer support: Developing a model for promoting health literacy (COPES) project on developing a theoretical model indicated against a need to identify a comprehensive sample of study reports. Instead the review team chose to prioritise relevance to the commissioners (i.e. research relevant to the National Health Service (NHS)), conceptual richness and contextual thickness. The COPES review was particularly challenging given an absence of consensus regarding either the scope or terminology of community engagement and peer support. In addition health literacy is a comparatively recent term, previously subsumed within broader concepts of health education and health promotion. As a consequence very few references included all three concepts even using an exhaustive list of synonyms and subject terms. Not only was study identification problematic but the resultant “I’ll know it when I see it” characteristic succeeded in transferring much of the retrieval effort from the search specialist to the topic experts. The review question was articulated using the Context-Intervention-Mechanism(s)-Outcome(s) (CIMO) framework (Table 3), a variant of the standard review formulation for a review question but one judged appropriate for realist synthesis questions [41].

**Table 3:** **Review question defined using CIMO framework**[41]

**Context**	UK or Developed Countries Health and Social Care
**Intervention(s)**	Peer Support and Community Engagement
**Mechanism**	[To be determined from subsequent exploration]
**Outcome**	Health Literacy

## Methods

An initial broad based search was conducted using an exhaustive list of peer support concepts (based on a previous review [42]) combined with (Health Promotion OR Health Education OR Health Literacy). Searches were conducted across PubMed, Web of Science and Scopus for the period January 1997-December 2012. Results were limited to English Language. A total of 14,488 references were found, reduced to 6,864 after elimination of duplicates.

All references were sifted by the review team using an Excel spreadsheet and drop down categories for coding for explicit mentions of peer support, community engagement and health literacy. Inconclusive records were referred for retrieval of full text. This original set of 455 references constituted the original sampling frame for the review. A two-layered approach was then used for all relevant records – 39 UK records were marked for prioritization with a further 416 non-UK studies being kept in a holding file.

The 39 UK articles reflected community engagement and peer support across a wide range of client groups. What was immediately apparent was the arbitrariness of retrieval and subsequent inclusion of references based only on title, abstract and keywords. Keywords were revealed as a “blunt instrument” in seeking to identify conceptual development, particularly when such concepts are emergent. The team identified several instances where one or more reports of an initiative had been retrieved, or subsequently coded as relevant, when equally relevant reports from the same study had been omitted or subsequently excluded. For example, a journal article entitled “Does bar-based, peer-led sexual health promotion have a community-level effect amongst gay men in Scotland?” [43] contains both peer education and community engagement concepts. However a related article, “Good in parts: the Gay Men's Task Force in Glasgow--a response to Kelly” [43], only labels the peer education concept. In this same article the community engagement concept is neither clear from the title nor the abstract. In such a case a review team would wish to be able to judge the project as a whole as being eligible or not. It would be a cause for concern if one report of a study resulted in the project being considered relevant and yet another report of the same study led to that same project being excluded. Once the project, as described in one particular paper, passes the requirements for inclusion, on the basis of relevance, this should, in the interests of consistency, open the door for inclusion to earlier and subsequent reports associated with the same project.

**Figure 1:**
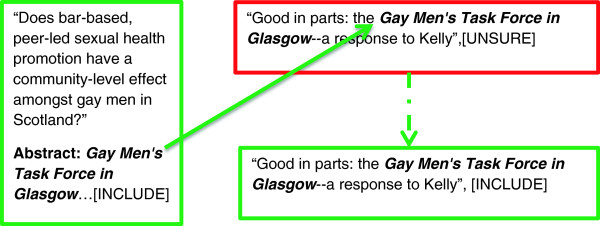
**Inclusion by affirmation.** Legend: In this case the inclusion of paper “Does bar-based, peer-led…” within the review modifies an earlier “Unsure” verdict for “Good in Parts”, based on a reading of Title and Abstract only. “Good in Parts”, as a sibling report to “Does bar-based, peer-led…” is now independently affirmed for inclusion within the review.

The team decided to combat the perceived inconsistencies associated with project inclusion by using a cluster approach. Included references identified from the topic-based bibliographic search, retrieved using keywords, became gatekeepers for additional references by association or referral. A previously missed or wrongly excluded reference might receive a further chance for inclusion by being “vouched for”, bibliographically speaking, by a sibling study that had already been included (Figure 1).

Eight UK-based projects were identified as candidates for a study cluster approach. One project, the Glasgow Gay Men’s Task Force (GMTF), was nominated as an initial case study for developing a methodology for cluster searching. This was the project for which the information specialist, a member of the review team, was simultaneously involved in extracting subsequent data. The cluster methodology, once developed appropriately, would then be extended to the other projects to identify study clusters. Reasons for selecting the GMTF study were not methodological. The GMTF study was typical of the other projects in comprising an index citation that could be linked through berrypicking approaches to sibling (directly related) and kinship (theoretically linked) literature.

### The Gay Men’s task force case study

The initial reference (“key pearl” or “index citation**”**) (Table 2), identified from the sift process, was “Does bar-based, peer-led sexual health promotion have a community-level effect amongst gay men in Scotland?” [43] (See Additional file 2 Directly Relevant Cluster References). The title contains explicit mentions of community, peer support and health promotion (as a proxy for health literacy). It therefore met all the review inclusion criteria.

#### In search of context

The information specialist started by checking the reference list of this key pearl citation for further references by the lead author. This reference checking identified two journal articles [44,45], three book chapters [46-48] a Web-based process evaluation [49] and two manuscripts under various stages of preparation [50] and [Flowers P, Frankis J, Hart G: *Experiential aspects of peer education in gay bars, Unpublished*]. Additional file 2 Directly Relevant Cluster References summarises items retrieved in this manner. Four [46-49] of these eight references, plus an unpublished manuscript [Flowers P, Frankis J, Hart G: *Experiential aspects of peer education in gay bars, Unpublished*], would not have been retrieved through searching of bibliographic journal-centric databases.

From the key pearl citation an author search on the Reference Manager database identified one additional reference [51] and confirmed full publication details of the In Press citation [50]. Both references had previously been excluded because they did not explicitly mention community engagement. Unlike the key pearl citation both these references used “Gay Men’s Task Force” in their title providing a search phrase for further Google searching. The next stage therefore involved identifying the lead author’s Web page on Google which established a full publications listing plus an up-to-date contact email. A second search, to identify the key pearl citation on Google Scholar, retrieved 35 references citing the key pearl citation. It also yielded the full text of citing articles. Noticeable was a Commentary by a team member from the same Unit as lead author but not otherwise explicitly connected with the GMTF Project [52]. The information specialist contacted the lead author and elicited three additional references (one Book Chapter, although already identified; and two tangential references from the GMTF Project) [53,54]. The former of these references [54] was particularly useful from a theoretical viewpoint suggesting the importance of locale or place when planning a community-based intervention targeting gay men. Contact with the author also established that the Submitted Article [Flowers P, Frankis J, Hart G: *Experiential aspects of peer education in gay bars, Unpublished*] had never been published. Unfortunately the lead author was unable to supply a copy of the manuscript draft.

At this point, having identified 14 items associated with the Project, the team could have considered that the context for the Glasgow Gay Men’s Task Force was sufficiently “thick” for analysis. Noticeably a high proportion of items in this cluster were not peer-reviewed journal articles with book chapters and a Web-based process evaluation among the items for inclusion. This confirms the limitations of topic-based bibliographic searches with respect to contextual information. Also supplementary channels, such as Google, Google Scholar and contact with authors, served to supply information that was otherwise unavailable. Useful findings, obtained through these channels, included (i) that a cited item had not subsequently been published, (ii) background project information with no explicit link to a study identifier and (iii) a related commentary by associated authors, but previously unrecognizably so as it excluded the author of the key pearl citation.

In truth this first stage of the CLUSTER procedure does not claim to be particularly innovative, certainly in terms of the techniques used. Many of these techniques are used to follow up initially included studies in many different types of systematic reviews. However the procedure uses three points of access (Authors, Citations and Project Names) to identify subsequent contextual information and, in contrast to previous examples, is documented in a systematic stepwise fashion. The thoroughness of this systematic approach is prerequisite to the subsequent, more innovative, steps associated with identification of theory.

#### In search of theory

Typically a review team is not able to explain how an intervention works simply from a thick description of context, whether located in a single study or in a cluster of studies. The team will also seek information on the theoretical basis for the intervention and to understand context as an explanatory variable( i.e. why an intervention works well in one setting but not well, or even not at all, in another). The first of three further lines of inquiry that may prove fruitful is to explore the theoretical “heritage” of the project.

### Unearthing “hidden” theory

Many of the previously-mentioned procedures to enhance contextual thickness utilize, and most notably, systematize, existing search techniques. The distinctive and innovative contribution of the CLUSTER method can be more readily determined in relation to the identification and subsequent investigation of theory. Examination of the full text of the key pearl citation and subsequent reports of the GMTF Project revealed passing citation of the Diffusion of Innovations Theory [55]. Such a finding led to two supplementary strategies. First, a search for “Diffusion of Innovations” on the reference management database for the community engagement project revealed other articles, whether initially included or excluded, that referenced the same theory. These articles suggested Diffusion of Innovations Theory as an explanation for how peer educators contribute to health literacy. This new iteration established a previously undiscovered commonality with another UK cluster; a project (ASSIST) examining adolescent peer support in schools to counter smoking [56]. Such a strategy enabled cross-case comparison [57], not previously apparent as a strategy for analysis. Furthermore had this project not already been included in the nominated UK clusters for the community engagement review this discovery could have informed further theoretical sampling and selection of additional clusters for analysis. Finally searches on Google Scholar for “Diffusion of Innovations” combined with the term “AIDS” helped to identify a theoretical paper entitled *Diffusion of innovations and HIV/AIDS*[58]. This paper analyzed why similar approaches citing this theory are successful in some circumstances and not in others.

Generally, journal articles may be limited as a source of conceptual development. Concepts may need to reach a particular state of realisation before being deemed worthy of publication in a peer-reviewed journal, whether this reflects the caution of the author, the inertia of the peer review community or, perhaps, a combination of both. As a consequence “qualitative publication bias” may exist in the form of a “time lag” so that, at any particular point in time, peer reviewed journals imperfectly, or incompletely, capture the current state of development of an emerging concept within a research community.

#### Identifying further information of potential relevance

The two final lines of inquiry, particularly when trying to explain why an intervention works in one setting but not in another, are (i) antecedent projects and (ii) similar contemporary projects. Manual checking of references for all studies in the cluster was used to reveal that project antecedents for the GMTF Project lay with two U.S.-based clusters of studies: the MPowerment Project [59-61] and highly-cited papers by Kelly and colleagues [62-64] (the “Kelly” mentioned in one of the titles above [51]). In addition a London-based project, the 4 Gyms Project, was linked as a contemporaneous UK-based study [65-67]. Identification of these three related projects led to two further strategies (Additional file 3 Identifying Wider Explanations of Theory and Context). First, citation searches for these three antecedent studies, prioritizing co-citations between projects, revealed a plethora of AIDS peer education studies, particularly in the developing world, drawing on the Diffusion of Innovations Theory. Finally, combining the project names or Lead Investigators for the GMTF and the MPowerment Project (because of topical proximity), for the GMHT and the 4 Gyms Project (because of the shared UK context) and for the GMTF and the ASSIST Project (because of their UK context and use of Diffusion of Innovations Theory) also yielded interesting insights. For example searches of Google and Google Scholar combining Flowers (Lead Author – GMTF) and Elford (Lead Author- 4 Gyms Project) identified a key article analyzing not just these projects but several other projects already present within our review UK clusters [68].

In contrast to the GMTF study, the two U.S. based studies were considered effective. This discrepancy in findings has led to commentaries reviewing all three projects, attempting to explain such differences, including a commentary by Kelly himself [69]. Such an insider perspective helps the review team to identify and explain any success or failure attributable to how the intervention was delivered or to its context.

## Results

By adapting techniques described by Bates (e.g. Reference chaining, follow-up of Author names) [16] in a systematic way the review team has grown an evidence base from an initial single included reference. Fourteen related project reports, thirteen available to the team, have enhanced the thickness of contextual data. However cluster searching does not only exploit the descriptive value of an expanded dataset. It also broadens the idea of “relevance” to include theoretical contributions and the explanatory power of the success or failure of similar studies (conceptual richness). Returning to our original Reference Manager database to search for “Diffusion of Innovations” Theory identified 49 studies including studies rejected by the initial sift. Revisiting initially rejected references evokes the berrypicking philosophy whereby information, initially rejected as irrelevant, subsequently becomes important. Establishing a link with Diffusion of Innovations Theory (i.e. “relatedness”: Additional file 3 Identifying Wider Explanations of Theory and Context) also led to supplementary searches examining the Diffusion of Innovations theory within the context of HIV/AIDS. Again the review team could not have identified *ab initio* either the significance of HIV/AIDS peer education as a context nor Diffusion of Innovations as a theory.

Similarly links to earlier U.S. studies, contemporary U.K studies and subsequent studies from the developing world were not identified at the start of the project. Four such projects (Mpowerment, 4 Gyms, ASSIST and the Kelly studies) offered further comparative analysis of factors relating to success and failure of the intervention. Searching for these projects individually and in conjunction, using (i) citation searching, (ii) author searching and (iii) searching by project name, yielded further richness.

## Discussion

### Towards a cluster searching methodology

This individual case study, presented using a narrative approach, suggests that cluster searching may be both practicable and desirable as a technique for harvesting rich and thick data. Such data can prove valuable when integrating quantitative and qualitative evidence and, specifically, in supporting realist synthesis. A recent realist review [70] independently utilizes a cluster-based approach to enhance the richness of data. The authors identified “23 partnerships, collectively composed of 276 documents, including peer-reviewed and non-peer-reviewed publications and websites”. Noticeably, however, this other review did not use a systematic approach to identify its clusters. Contact with authors was the single method used in this instance. Contact with authors may help to identify most, if not all, papers directly associated with a named cluster. However it would not reveal either additional papers invoking theory or related projects with a common provenance.

Berrypicking requires the searcher either to have a very clear idea of the relevance of individual items as encountered or to work in tandem with the subject expert. Asynchronous approaches, whereby the searcher completes the searches independently and sends the results of the search to a subject expert (as for most topic-based bibliographic searches), are not typically accommodated by these interactive elements of berrypicking. However synchronous approaches, where both searcher and subject expert work side-by-side, may be prohibitive, in terms of both time and availability, for most types of iterative searching. In contrast identification of study clusters is potentially a more objective task requiring less subject knowledge and harnessing the same cues that information specialists routinely use in their role. Where a searcher and subject expert have agreed an overall searching strategy this may obviate the need for the subject expert to be present when the searching itself takes place. Development of a formalised and agreed set of procedures for searching for study clusters would ensure that berrypicking can preserve its flexible and iterative nature whilst being “reinvented” as a systematic and rigorous component in the literature searching toolkit. As Sandelowski and Barroso observe:

“the searcher wanders through the information forest, changing direction as needed to follow up on various leads and shifts in thinking. The key is to keep track of and account for these shifts” [18].

A formalised and agreed set of procedures for searching for study clusters would also help to ensure a greater level of consistency in how the searcher follows leads and pursues changes in direction, as prompted by review of retrieved results.

A suggested procedure for cluster searching, generalized from the individual case study, is presented in Table 4. It may be helpful for information specialists, in particular, to observe the strict sequence of the thirteen stages of the procedure. However the essence of the cluster search method is embodied in the CLUSTER mnemonic (Table 5).

**Table 4:** Suggested generic procedure for cluster searching

**Steps to enhance exploration of context**		
Step	Procedure	Source(s)
1	Identify at least one key “pearl” citation, agreed through consensus by the review team	Preliminary Literature Search of bibliographic databases
2	Check Reference list for any additional relevant citations by the Authors	Full text of pearl citations
3	Recheck for additional relevant records by the Authors	Reference management database
4	Search for lead author (and other authors as appropriate). Seek to identify Contact email, Publications list, Institutional repository	Google
5	Conduct citation searches on key pearl citation (and other publications as appropriate)	Web of Science/Google Scholar
6	Conduct searches on project name/identifier (if available)	Google Scholar
7	Make contact with Lead Author (particularly regarding related publications, unpublished articles, reports, book chapters etcetera)	Personal Web pages
**Steps to enhance identification of theory**		
Step	Procedure	Source(s)
8	Follow up key pearl citation and other cluster documents for citation of theory	Full text of pearl citations
9	Recheck for mentions of Theory in titles, abstracts, keywords	Reference management database
10	Optionally, perform iterative searches for theory mentioned in combination with Condition of Interest	Original set of bibliographic databases
**Steps to broaden the search to other relevant information**		
Step	Procedure	Source(s)
11	Follow up key pearl citation and other cluster documents for citations to project antecedents and related projects	Full text of pearl citations
12	Conduct named project and citation searches for relevant projects identified from cluster documents	Google Scholar/Web of Science
13	Seek cross case comparisons by combining project name/identifier for cluster with project name/identifiers for other relevant projects	Original set of bibliographic databases

**Table 5:** CLUSTER mnemonic for components of cluster search methodology

**Element**	**Procedural steps (See Table**2**)**
**C**itations	Step 1
**L**ead Authors	Steps 2-4
**U**npublished materials	Step 7
**S**cholar searches	Steps 5-6
**T**heories	Steps 8-10
**E**arly Examples	Step 11
**R**elated Projects	Steps 12-13

Our proposed CLUSTER methodology utilizes most of the six procedures suggested by Bates [16] [Table 5]. Of particular importance is footnote (or reference) chasing which is used in three different ways: to identify papers by the project team (Step 2), to identify relevant theory (Step 8) and to identify project antecedents and relevant related projects (Step 11). Citation searching, harnessing the powerful facilities of Google Scholar (and Web of Science if available) is utilised to search for references citing the cluster documents (Step 5) or citing relevant projects (Step 12). A variant of area scanning (in this case, using the author’s web page (Step 4) to identify related publications) updates the physical equivalent suggested by Bates [16]. The CLUSTER procedures complement the topic-based searches used earlier in the review process (which correspond to Bates’ abstracting and indexing searches [16]), previously the most developed of the six methods. Searching of abstracting and indexing sources is also employed to follow up a specific theory (Step 10). Author searching is used to identify cluster documents relating to the project of interest (Steps 3 and 4) as well as clusters of documents associated with related projects. In fact the only search procedure mentioned by Bates [16] not included in the CLUSTER method is browsing through journal runs. Arguably purposively searching by project name or identifier as a retrieval key (Steps 12 and 13), supplants Bates’ more serendipitous browsing of journal runs [16].

Finally, experience from generating, and more importantly, evaluating the yield from cluster searching may help reviewers to reconceive “richness” as a systematic amalgamation of “thickness”, as previously identified by Denzin [34] and additional layers of conceptual richness relating to theoretical and conceptual contribution, an understanding of wider contextual effects and interpretive power to support inference.

### Limitations

The principal limitation of the CLUSTER methodology is that it has been explored in relation to one case study cluster. We do not know whether the total of research outputs for a typical project compares unfavourably with the number identified for the GMTF. However the number of outputs from a project is not the sole determinant of the value of the CLUSTER procedure. If the overall purpose of a review is to achieve an in-depth understanding of the context and implementation of an intervention then arguably even one additional report, such as a book chapter or Web document, can contribute to this objective. At least one additional cluster, the ASSIST Project [56], spawned similar richness of reporting and analysis as the GMTF. Indeed, if few documents are retrieved from stages 1–7 of the CLUSTER procedure this would make the additional stages 8–13 relatively more valuable. The resultant “external” frames of reference and cross case comparison may compensate for the paucity of “internal” data.

Another limitation is that the opportunistic nature of this investigation did not allow detailed record-keeping on the amount of time taken to identify each additional relevant item. However cluster searching should not be considered an alternative to topic-based searching. Rather the CLUSTER procedures are supplementary, complementing deficiencies or omissions from topic-based searches. Several factors will determine whether it is cost-effective to utilize the CLUSTER procedures. Considerations include the complexity of the research question, the complexity of the intervention, the prior conceptual development of a topic, the precision of the search terms and the overarching purpose of the synthesis. Indeed a searcher could briefly “triage” the topic using only the procedures associated with the key pearl citation to predict the likely value of the CLUSTER procedures. The searcher would retrieve the full text of the key pearl citation and identify (i) any references by the authors, (ii) any citations to theory and (iii) any citations to related projects. At the very least an abbreviated CLUSTER procedure would offer a validation check for the topic-based search. A further citation search on Google Scholar could speedily establish how influential the key pearl citation has been. The review team could then make an informed judgement on the added value of performing the full CLUSTER procedures from Table 4.

Cluster searching offers a greater potential contribution to realist reviews, qualitative syntheses of complex interventions or those reviews where implementation-related issues figure prominently. Further investigation is required to establish whether capture of additional reports merely signals duplicate publication or whether it yields additional data [71]. Even where “salami-slicing” has occurred, if there is minimal overlap, additional reports will still yield useful data, not previously available to the review. Our experience revealed that, rather than representing “rehashes” of peer-reviewed journals, book chapters may indeed prove valuable. For example one book chapter identified particular flaws in viewing “men with HIV” as a homogenous group [47], flagging the existence of significant subgroups and tensions between members of each. Nevertheless further work needs to examine the relative contribution of each source of richness to a final synthesis and our understanding of the intervention, working outwards from the sibling studies to more distantly related studies. A qualitative sensitivity analysis, would demonstrate the extent to which the full CLUSTER approach might be considered worthwhile [72].

A further consideration is that an approach that essentially recreates a network or web of related studies on the basis of “aboutness” or “relatedness” runs the risk of missing alternative, but relevant, hypotheses, research traditions and theories. To a certain extent this is not a limitation of cluster searching *per se* but will depend on how well included studies are reported and interpreted. Procedures that optimize identification of the “disconfirming case” will, by analogy, to be of relevance here [73]. In addition, techniques associated with qualitative synthesis require reviewers to attempt to refute them with alternative interpretations. One particular strength of the CLUSTER method relates to the fact that theories are identified “forensically”, from the actual evidence base for a project rather than being “magicked” via external interpretation from the review team. Furthermore the CLUSTER method provides three opportunities to identify competing theories within such a cluster; from reports from the project itself, from related projects and from overview papers reviewing several related projects. The risk of only partial theoretical insights, although undeniably present, is arguably reduced in contrast to serendipitous methods for identifying theory.

Although cluster searching is being advanced as a potential method for extending and enhancing the identification of relevant data for use in a systematic review we must acknowledge that much remains to be explored with regard to the characteristics of reports located within a particular study cluster. For example, we do not know whether quantitative studies are more likely to be published before or after associated qualitative studies. Neither do we understand where and when process evaluations or economic evaluations enter the picture, or what a typical interval is between sibling publications. We know very little on how securely different types of study are “linked” in terms of number of authors in common, cross citation (i.e. to each other), citations in common or study identifiers. In addition we do not really know how plentiful sibling reports are, how easy they are to access or obtain and how useful they are for the synthesis once obtained. Specifically, with regard to study identification, we do not know how easy it is to identify sibling reports. Nor do we have a clear idea what the best search procedures are with which to retrieve sibling reports. We cannot determine whether each project requires an idiosyncratic process of study identification or whether projects could benefit from a generic approach to sibling studies. Pawson describes “the prolonged and repetitive agony of locating appropriate primary materials” [74]. Pawson’s comment is echoed more equivocally by Hughes who describes “the false trails, the frustrations, the subtle shifts in thinking, or the surprises, satisfactions and rewards, which characterised the whole experience” [21].

The CLUSTER procedures systematize and formalize existing processes and assign a clear responsibility for supplementary searching. For example despite widespread agreement that “references should be followed up” there is a current lack of clarity on whether follow-up is the responsibility of the reviewer or the information specialist. Considerable variability currently exists around how rigorously follow-up of references is implemented and documented by different review teams.

## Conclusions

In view of the acknowledged limitations of using a single case study this article stops short of suggesting that CLUSTER should be a standard component of study identification procedures for all systematic reviews. Nevertheless CLUSTER does possess relative advantage over current methods; it retrieves items known to be elusive to topic-based search procedures, it yields data otherwise lost to a review project, it establishes a basis for theoretical analysis and for cross case comparison. Perhaps most importantly it establishes a transparent procedure for berrypicking techniques within the rigorous context of a systematic review. CLUSTER procedures can easily be documented in standard tables – as used to document this case study (See Additional files 2 and 3), within a four or five page appendix. Far more important than simply documenting this otherwise messy and iterative process is the fact that, by following the CLUSTER procedures, an information specialist would achieve a manifestly more rigorous, consistent and high quality output.

In addition to the added value of additional studies, there are good qualitative reasons for suggesting use of the CLUSTER procedures. First, an understanding of theoretically informed complex interventions is critical to establishing their effectiveness. Including a formal search process that identifies citations linking practice to theory is one means for encouraging such a connection. Similarly the cross case comparison embodied in the CLUSTER methodology supports an overarching principle that systematic review and evidence synthesis consolidate knowledge from research. Of course, such advantages may not be realizable for every review and a significant proportion of reviews may be constrained to identification of directly related cluster studies (steps 1–7).

Study identifiers are of particular value in facilitating study identification and retrieval. Memorable and distinctive project names provide an effective retrieval key for related reports. With the popularization of the International Standard RCT Identification number (ISRCTN) [75], comes the prospect of improved retrieval of associated articles. However enhanced retrieval by ISRCTN only relates to reviews where the original key pearl citation is a randomized controlled trial and, as with the grant or project identification numbers used by commissioners of research, requires that researchers consistently attribute their research. For this reason systematic review teams in general, and information specialists in particular, should advocate use of identification numbers and/or memorable names in web pages, reports and manuscripts for submission.

Finally, one attraction of the CLUSTER procedure is that it offers a systematic way to identify useful contributions to understanding of a project without requiring topical knowledge or making definitive judgements on relevance. Levels of “aboutness” [76], relating a key pearl citation to other documents, are established by informational cues that an information specialist typically has been trained to identify, namely author names, project identifiers and citations of theory or related work. An information specialist can establish the potential significance of any subsequent items identified for the review through tangible markers such as numbers of citations and the presence of co-citation. Having produced a brief structured report on the results of the CLUSTER procedures the information specialist could hand this over to the review team and its topic experts for definitive judgements on (i) whether other identified papers truly belong in the project cluster and (ii) the degree of relatedness of other cited projects. Indeed the phased nature of the procedure offers the possibility for periodic review of items being retrieved and evaluation of whether to continue the process. Following the CLUSTER procedure will also help the information specialist to communicate a systematic approach to supplementary searching and maintain involvement in the review team beyond the initial topic-based bibliographic searching phase.

## Competing interest

The authors declare that they have no competing interests.

## Author contributions

AB carried out the initial cluster searches and drafted the manuscript. AB and JH conceived the study, and all authors read and commented on the drafts and read and approved the final manuscript.

## Pre-publication history

The pre-publication history for this paper can be accessed here:

http://www.biomedcentral.com/1471-2288/13/118/prepub

## Supplementary Material

Additional file 1 Schema of Cluster Documents. Click here for file

Additional file 2 Directly Relevant Cluster References. Click here for file

Additional file 3 Identifying Wider Explanations of Theory and Context. Click here for file
