# Supplementary material for: Towards a methodology for cluster searching to provide conceptual and contextual “richness” for systematic reviews of complex interventions: case study (CLUSTER)
Source: BMC Med Res Methodol. 2013 Sep 28;13:118. doi: 10.1186/1471-2288-13-118 (PMC3819734; doi:10.1186/1471-2288-13-118)
Supplement: Additional file 1 — Schema of Cluster Documents. [file 1471-2288-13-118-S1.docx]

Additional file 1 - Schema of Cluster Documents

| **Sibling Papers [30]** | | **Kinship Studies** | | **Distant Relations** | |
| --- | --- | --- | --- | --- | --- |
| Definition | Examples | Definition | Examples | Definition | Examples |
| Papers conducted by same authorial team or describing same phenomenon of interest | Qualitative reports of theory building, papers reporting pilot studies to test relationships for proposed intervention, reports of tests of intervention effects, economic evaluations and papers reporting implementation and roll out. | Studies that relate to original phenomenon of interest across one or two variables e.g. use of theory, setting of intervention, main components etcetera | Studies that further develop theory across other settings, people, situations; pragmatic studies that test hypothesis in different environments; studies that replicate core components of the intervention but vary supplemental components; and long term follow up studies for each of these. | Studies detached from phenomenon of interest so as not to overtly contribute to theory development but which contain explanatory data relevant to the context/phenomenon of interest | Synthesis studies of common theory, intervention, or context offering variability for comparison and contrast. May represent a “best fit” rather than a match. |
| **Data Sources:**  Reports that precede journal publication, journal articles, long term follow up studies and subsequent critiques and commentaries | | **Data Sources:**  Citing documents and cited documents; documents sharing common citations e.g. to a theory, predecessor study | | **Data sources:**  Discussion sections of papers and syntheses, theoretical overviews | |
