# Supplementary material for: Towards a methodology for cluster searching to provide conceptual and contextual “richness” for systematic reviews of complex interventions: case study (CLUSTER)
Source: BMC Med Res Methodol. 2013 Sep 28;13:118. doi: 10.1186/1471-2288-13-118 (PMC3819734; doi:10.1186/1471-2288-13-118)
Supplement: Additional file 2 — Directly Relevant Cluster References. [file 1471-2288-13-118-S2.docx]

Additional file 2 - Directly Relevant Cluster References

| **Key “pearl” citation** |
| --- |
| “Does bar-based, peer-led sexual health promotion have a community-level effect amongst gay men in Scotland?” (Published Article) [43] |
| **Reference Checking from key “pearl” citation [Identified from Author Surname]** |
| Evidence and the evaluation of a community-level intervention: researching the Gay Men’s Task Force initiative (Book Chapter) [47]. |
| Gay Men’s Task Force: the impact of peer education on the sexual health behaviour of gay men in Glasgow. (In press citation) [51] |
| Homosexual men’s HIV-related sexual risk behaviour in Scotland (Journal Article) [45] |
| `Everyone on the scene is so cliquey’: are gay bars an appropriate social context for a community based peer led intervention? (Book Chapter) [48] |
| Experiential aspects of peer education in gay bars (Submitted manuscript – never published) [52] |
| Gay Men’s Task Force: Preliminary Evaluation of Service Delivery (Process evaluation – Web document) [50]. |
| Identities and gay men’s sexual decision making (Book Chapter) [49] |
| Gay men and HIV/AIDS risk-management (Journal article) [46] |
| **Other Reference Manager database records [Identified from Author Surname]** |
| Good in parts: the Gay Men's Task Force in Glasgow--a response to Kelly (Published Authors Reply) [44] |
| The Gay Men's Task Force: the impact of peer education on the sexual health behaviour of homosexual men in Glasgow (Published Article) [51] (Confirmed details of In Press Citation) |
| **Google searching “Paul Flowers”** |
| Contact email |
| Web Page including List of Publications |
| Link to Institutional Repository |
| **Google Scholar searching “Gay Men’s Task Force”** |
| 37 references (including some full text) plus |
| Correspondence: HIV optimism does not explain increases in high-risk sexual behaviour among gay men in Scotland (Letter from other researchers) [53] |
| **Contact with Lead Author** |
| Revealed that Submitted article did not get published |
| Identified three relevant references ([48] plus two others): |
| `The bars, the bogs, and the bushes': the impact of locale on sexual cultures (Published Article) [54] |
| Constructing Sexual Health: Gay Men and ‘Risk’ in the Context of a Public Sex Environment (Published Article) [55] |
|  |
