# Supplementary material for: Towards a methodology for cluster searching to provide conceptual and contextual “richness” for systematic reviews of complex interventions: case study (CLUSTER)
Source: BMC Med Res Methodol. 2013 Sep 28;13:118. doi: 10.1186/1471-2288-13-118 (PMC3819734; doi:10.1186/1471-2288-13-118)
Supplement: Additional file 3 — Identifying Wider Explanations of Theory and Context. [file 1471-2288-13-118-S3.docx]

Additional file 3 - Identifying Wider Explanations of Theory and Context

| **Following Up Theory** |
| --- |
| Looking for References to Theory in Included Cluster Articles |
| Searching for mentions of the Theory in the Review Database |
| Iterative Searches for Theory combined with the Condition of Interest |
|  |
| **Identifying Project Antecedents** |
| Reference checking |
| - MPowerment Project |
| - Kelly et al Studies |
| - 4 Gym Project |
|  |
| **Identifying Related Projects** |
| Citation searching |
| - Citation searches for MPowerment Project |
| - Citation searches for Kelly et al Studies |
| - Citation searches for 4 Gym Project |
|  |
| Combining Project Names |
| - GMTF and MPowerment |
| - GMTF and 4 Gym Project |
| - GMTF and ASSIST |
|  |
